# Clinical and Functional Differences Between Mexican Youth at Clinical High Risk for Psychosis and With Familial High Risk

**DOI:** 10.3389/fpsyg.2022.911030

**Published:** 2022-07-04

**Authors:** Lourdes Nieto, Tecelli Domínguez-Martínez, Mauricio Rosel-Vales, Ricardo Saracco-Alvarez, Cesar Celada-Borja, María Luisa Rascón-Gasca

**Affiliations:** ^1^Division of Epidemiological and Psychosocial Research, Center for Global Mental Health Research, National Institute of Psychiatry Ramón de la Fuente Muñiz, Mexico City, Mexico; ^2^Schizophrenia Clinic, National Institute of Psychiatry Ramón de la Fuente Muñiz, Mexico City, Mexico; ^3^Department of Clinical Research, National Institute of Psychiatry Ramón de la Fuente Muñiz, Mexico City, Mexico; ^4^Division of Epidemiological and Psychosocial Research, Department of Social Sciences in Health, National Institute of Psychiatry Ramón de la Fuente Muñiz, Mexico City, Mexico

**Keywords:** clinical high risk for psychosis, at-risk mental states, family risk, unaffected relatives, early psychosis

## Abstract

Few studies have explored the differences in clinical psychopathology between youth at high risk for psychosis and those at familial high risk for psychosis. This study seeks to describe and compare the sociodemographic, clinical, and functional characteristics of At-Risk Mental State (ARMS) for psychosis youth and those with a first- or second-degree relative with psychosis (Familial High-Risk: FHR) in a Mexican sample. Twenty-one ARMS individuals and 21 with FHR were evaluated for sociodemographic characteristics, psychopathological symptoms, and functional impairment. ARMS individuals were significantly younger, had fewer years of schooling, and were more likely to be male than those in the FHR group. Groups did not differ as regards marital status or occupation. The ARMS group showed greater severity of prodromal symptoms, schizotypal personality traits, and general psychopathology than the FHR group. In addition, they reported more premorbid adjustment deficit from early adolescence than the FHR group. Current overall social and role functioning was significantly lower in the ARMS group. Findings are consistent with ARMS studies from other countries. First- or second-degree relatives of patients with psychosis should be considered a vulnerable group as they display several symptoms of general psychopathology and may experience social adjustment problems in their adult lives. The lack of early detection and intervention psychosis programs in Mexico underlines the need to prioritize the development of preventive strategies to help close the care gap.

## Introduction

Psychosis is a complex syndrome characterized by the presence of a broad range of symptoms and functional impairment (Krabbendam et al., 2004; Guloksuz and van Os, 2018; Althwanay et al., 2020) that typically emerges in late adolescence, disrupting the transition into adulthood, causing impairments in all areas of life, and generating major personal, social, and economic costs (Kennedy et al., 2014; de Girolamo et al., 2019).

The onset of a psychotic episode is often preceded by a prodrome, characterized by a range of nonspecific signs and symptoms, and attenuated or subthreshold psychotic symptoms that cause a change in premorbid functioning (Addington et al., 2019; Carrión et al., 2021). The psychosis prodrome provides a unique opportunity to identify psychosis onset mechanisms and test early intervention strategies (Woodberry et al., 2016). The possibility of prospectively monitoring those clinically at high-risk of psychosis was the catalyst for worldwide research pursuing the elusive goal of preventing the onset of a psychotic disorder or at least helping to improve the course of the disorder and reduce its long-term impact (McGorry et al., 2021). The early detection and intervention paradigm is based on reducing the duration of untreated psychosis (DUP), since prolonged DUP is associated with neurotoxic damage, greater severity of psychotic symptoms, low functioning, and poor quality of life (Marshall and Rathbone, 2011).

Individuals at risk for psychosis are typically identified by risk indicators and clinical signs and symptoms that point to an increased likelihood of developing a psychotic disorder (Woodberry et al., 2016). According to a review by Woodberry et al. (2016), these individuals are identified by age (typically those aged 12–35) and clinical characteristics (primarily new or worsening attenuated psychotic symptoms) suggestive of a psychosis prodrome. Since the majority will not transition to a diagnosable psychotic disorder (van Os and Guloksuz, 2017), these prospectively identified individuals are typically referred to as “clinical high-risk” (CHR), “ultra-high risk” (UHR), or as having “at-risk mental states” (ARMS; Yung et al., 2004; Woodberry et al., 2016).

The at-risk stage comprises a heterogeneous group of symptoms traditionally described during the prodromal phase of psychosis. Current standard definitions and operationalization yield three clusters of individuals: (1) people with attenuated positive psychotic symptoms; (2) those who have experienced brief intermittent episodes of frank psychotic symptoms lasting no more than a week, with spontaneous full recovery; and (3) those having a first-degree relative diagnosed with a psychotic disorder or meeting the criteria for schizotypal personality disorder. With a significant decrease in social functioning in all cases (McGlashan et al., 2001; Miller et al., 2003; Yung et al., 2004).

The most validated model to explain the etiology of schizophrenia-spectrum disorder is based on the interaction of environmental and genetic risk factors (Rasic et al., 2014; Radua et al., 2018). Youth with first- and- second-degree relatives with psychosis (familial high-risk, FHR) have an 8–12% and 3–4% lifetime risk of developing psychosis, respectively, (Sullivan, 2005). Poletti et al. (2021) recently found that more than 60% of UHR individuals had a family history of serious mental illness, while a third had at least one first-degree relative with psychosis. In a longitudinal study with FHR adolescents, Shah et al. (2019) observed that over 3 years, 8.3% developed a psychotic disorder, and 65% met the criteria for an Axis I disorder over the course of the study. In a community sample, Taylor et al. (2020) found that a family history of psychosis was a significant risk factor for psychotic symptoms, mood, externalizing, fear symptoms, and poorer functioning.

To date, only a few studies have directly compared UHR with FHR individuals, with most focusing on exploring differences in cognitive performance. A meta-analysis found that youth with FHR had deficits in several cognitive domains and executive function compared to youth without a family history of schizophrenia (Bora et al., 2014). Chu et al. (2019) reported that UHR and FHR individuals experienced largely comparable cognitive impairment that was midway between first-episode schizophrenia-spectrum disorder patients and healthy controls. Hou et al. (2016) found that impairments in processing speed, attention, working memory, and verbal memory exist in both UHR and FHR individuals. Moreover, FHR individuals had poorer performance than healthy participants. Additionally, there is evidence indicating that relatives of psychotic patients show levels of stress sensitivity that are midway between healthy controls and psychotic patients (Aiello et al., 2012).

Findings of a study comparing risk factors, psychopathology, and functioning between healthy controls and two groups of FHR individuals, one of which also met UHR criteria (FHR + UHR), showed that there was a trend for the FHR-non-UHR group to be midway between healthy controls and the FHR + UHR as regards symptoms and functioning, although differences were not always significant. In addition, the FHR + UHR group had significantly worse early premorbid childhood functioning than controls, whereas FHR-non-CHR participants and controls did not differ as regards earl premorbid childhood functioning (Stowkowy and Addington, 2013). Studying individuals at risk of developing psychosis, either because of genetic vulnerability (such as FHR) or because they present subthreshold psychotic symptoms (ARMS), provides a valuable opportunity to examine risk factors as they have few of the confounds associated with medication exposure (antipsychotic) and illness duration (Shah et al., 2019). Since psychopathology and help-seeking behavior in FHR individuals who do not meet UHR criteria remain poorly understood (Norman et al., 2007; Stowkowy and Addington, 2013), more research is required to explore demographic, clinical, and functional characteristics of FHR as compared to those of UHR, to better understand the combination of potential factors that could explain why some FHR or UHR go on to develop psychosis and others do not.

The aim of this study is therefore to describe and compare the sociodemographic, clinical, and functional characteristics of Mexican youth at-risk mental state (ARMS) for psychosis, and first- or second-degree relatives of patients diagnosed with a psychotic spectrum disorder (FHR) who do not meet ARMS criteria.

## Materials and Methods

The present study is part of a broader longitudinal study focusing on the early detection of psychosis conducted in Mexico City. Baseline assessments with complete data on relevant outcome measures were included in the current descriptive and observational study.

General inclusion criteria for the ARMS and FHR groups were (1) being aged 13–40; (2) an ability to understand the survey instructions and contents; and (3) having completed at least elementary school. The specific inclusion criterion for the ARMS group was meeting the criteria for at least one of the ARMS groups established by the Comprehensive Assessment of At-Risk Mental State (CAARMS; Yung et al., 2005). Specific inclusion criteria for the FHR group were having either a first-and/or second-degree relative affected by a psychosis spectrum disorder and not meeting the criteria for any of the ARMS groups established by the CAARMS.

General exclusion criteria for the ARMS and FHR groups were (1) intellectual disability; (2) significant head injury or current medical or neurological condition; (3) an organically based psychosis; (4) a lifetime diagnosis of a psychotic spectrum disorder according to the DSM-V (American Psychiatric Association [APA], 2013); or (5) meeting the criteria for a psychosis threshold as determined by the CAARMS.

### Sample

The study included 42 participants, classified into two groups. The first group included 21 individuals who met the ARMS criteria established by the CAARMS (Yung et al., 2005). The predominant diagnostic category was Attenuated Psychosis Syndrome (APS; 85.7%, *n* = 18), followed by the trait vulnerability of psychosis criteria (14.3%; *n* = 3). No participants met the criteria for Brief Limited Intermittent Psychosis Symptoms (BLIPS). At the time of the study, participants were under psychological and/or psychiatric treatment at the National Institute of Psychiatry (INPRFM) in Mexico City.

The second group of Familial High-Risk (FHR) comprised 21 first- or second-degree relatives (nine offspring and 12 siblings) of patients diagnosed with a psychotic spectrum disorder by their treating psychiatrists. None of the FHR met ARMS criteria. FHR participants were referred by the psychiatrists of their affected family members or through a psychoeducational program at the INPRFM designed for caregivers of people with mental illness.

### Measures

#### Demographic and Clinical Data

Demographic and clinical background information was obtained through a semi-structured interview.

#### At-Risk Mental State Criteria

ARMS criteria and symptoms were assessed by the CAARMS (Yung et al., 2005). The CAARMS is a 28-item, semi-structured interview developed to identify subjects at imminent risk of psychosis. It assesses seven symptom domains: positive symptoms, cognitive symptoms, emotional disturbance, negative symptoms, behavioral change, motor change, and general psychopathology (Yung et al., 2005). ARMS criteria are established if the severity, frequency, or duration of positive symptoms are below threshold levels for psychosis and are divided into three groups: (1) APS subgroup: the presence of subthreshold positive symptoms (either in frequency or intensity) during the past year; (2) BLIPS subgroup: the presence of episodes of frank psychotic symptoms in the past year, which spontaneously resolved within a week; or (3) vulnerability subgroup: having a schizotypal personality disorder or a family history of psychosis in a first-degree relative and having experienced a significant decrease in functioning in the past year (Yung et al., 2005).

#### Schizotypal Traits

The *Schizotypal Personality Questionnaire* (SPQ; Raine, 1991; Rabella et al., 2018; Marrero et al., 2020) is a 74-item self-report questionnaire with a yes (1 point) or no (0 points) answer format. It identifies the nine schizotypal personality disorder traits, according to the DSM-III-R (American Psychiatric Association [APA], 1980), and a schizotypal total score (range: 0–74). Higher scores represent a greater degree of schizotypal traits.

#### General Psychopathology

The *Symptom Checklist-90* (SCL-90; Derogatis, 1977; Lara et al., 2005) is a 90-item self-report on a five-point Likert scale that evaluates nine dimensions of psychopathology: somatization, obsession-compulsion, interpersonal sensitivity, depression, anxiety, hostility, phobic anxiety, paranoid thoughts, and psychosis. Higher scores indicate greater severity of symptomatology.

#### Premorbid Adjustment

*Premorbid Adjustment Scale* (PAS; Canoon-Spoor et al., 1982; López et al., 1996) is an interview-based rating system designed to retrospectively assess functioning in five psychosocial domains: sociability/withdrawal, peer relationships, school performance, school adaptation, and socio-sexual life. Each of these domains is assessed at four developmental stages: childhood (up to age 11), early adolescence (12–15 years), late adolescence (16–18 years), and adulthood (≥19 years), except that the socio-sexual domain is assessed after age 15. Ratings are expressed as decimals ranging from 0 to 1, with higher scores indicating lower levels of premorbid adjustment.

#### Social and Occupational Functioning

The *Social and Occupational Functioning Assessment Schedule* (SOFAS; Goldman et al., 1992) and the *Global Functioning: Social and Role Scales* (GF-Social and GF-Role; Cornblatt et al., 2007) were used to assess current levels of functioning.

### Procedure

The study was approved by the Research Ethics Committee of the INPRFM (Approval No. CEI-010-20170316) and adheres to the Declaration of Helsinki. Written informed consent was obtained from all participants, as well as from the parents or legal guardians of minors. Participants did not obtain any financial compensation for their participation.

### Statistical Analyses

Data were analyzed using SPSS 24.0 for Windows. Descriptive statistics were used to summarize the demographic and clinical characteristics of the sample. Duration of Untreated Illness (DUI) was defined as the time interval from the onset of non-specific symptoms to receiving the first specialized psychiatric and/or psychological treatment (Esterberg and Compton, 2012). Groups were compared using independent *t*-tests for continuous variables and chi-square tests for categorical variables. The effect size (Cohen’s *d*) was also analyzed according to the Cohen’s guidelines (small, *d* = 0.2, medium, *d* = 0.5 and large, and *d* = 0.8; Cohen, 1998).

## Results

### Demographic Data

Demographic data are shown in Table 1. Participants in the ARMS group were significantly younger and had fewer years of schooling than those in the FHR group. Most of those in the ARMS group were male, whereas in the FHR group, the majority were female. Groups did not differ in terms of marital status, occupation, or household type.

**Table 1:** Demographic characteristics of sample (*N* = 42).

	ARMS (*n* = 21)		FHR (*n* = 21)		Comparisons	
					*t(df)*	Effect size Cohen’s *d*
Age (years)	21.9 ± 5.8		29.1 ± 7.0		3.58^***^ (40)	1.1
Years of schooling	12.3 ± 3.3		16.1 ± 3.0		3.84^***^ (40)	1.2
	*n*	**%**	*n*	**%**	*X^2^(df)*	
Highest education level completed						
Elementary school	1	4.8	0		13.26^**^ (3)	
Middle school	9	42.9	2	9.5		
High school	8	38.1	5	23.8		
University	3	14.3	14	66.7		
Sex						
Male	16	76.2	5	23.8	11.52^***^ (1)	
Female	5	23.8	16	76.2		
Marital status						
Single/separated/divorced	19	90.5	14	66.7	3.53 (1)	
Married/cohabiting/dating	2	9.5	7	33.3		
Occupation						
Unemployed/not working	4	19.0	3	14.3	0.86 (2)	
Employed	8	38.1	11	52.4		
Student	9	42.9	7	33.3		
Household						
Living alone	0		2	9.5	6.58 (2)	
Partnered	2	9.5	6	28.6		
Family of origin	19	90.5	12	57.1		
Friends/roommate	0		1	4.8		

ARMS, at-risk mental state and FHR, familial high risk. Mean ± SD.

*** *p* < 0.001;

** *p* < 0.01.

### Substance Use, Current Use of Mental Services, and Treatment History

As shown in Table 2, in both groups, alcohol was the most used substance in the past 12 months, particularly in the FHR group. No differences between groups were found in cannabis or tobacco use, either as regards age of onset or use. There were no statistically significant differences between groups as regards current use of mental health services. However, significant differences were found in current psychiatric treatment. In the ARMS group, 42.9% were taking antidepressant medication, 38.1% antipsychotics, and 23.8% anxiolytic and/or anticonvulsant medication, whereas in the FHR group, only 4.8% were taking antidepressant medication. The current main reasons for consulting a mental health service in the ARMS group were psychosis-like experiences and depression or anxiety symptoms. Only 9.5% of participants with FHR used mental health services for depression or anxiety, fibromyalgia, or grief symptoms. In the ARMS group, the mean DUI was 118.7 weeks (SD = 143.1; Range = 6–522).

**Table 2:** Substance use, current use of mental services and treatment history of sample (*N* = 42).

	ARMS (*n* = 21)		FHR (*n* = 21)		Comparisons	Effect size Cohen’s *d*
	*n*	**%**	*n*	**%**		
Substance use						
Cannabis						
History of use (at least once): Yes	8	38.1	11	52.4	*X^2^* = 0.86; *df* = 1	
Use during the past 12 months: Yes	4	19.0	6	28.6	*X^2^* = 0.52; *df* = 1	
Age of onset	17.8 ± 5.0		18.7 ± 2.7		*t*(17) = 0.47	0.2
Alcohol						
History of use (at least once): Yes	20	95.2	18	85.7	*X^2^* = 1.10; *df* = 1	
Use during the past 12 months: Yes	11	52.4	15	83.3	*X^2^* = 4.17^*^; *df* = 1	
Age of onset	15.3 ± 4.9		14.7 ± 3.6		*t*(36) = −0.40	−0.1
Tobacco						
History of use (at least once): Yes	14	66.7	12	57.1	*X^2^* = 0.40; *df* = 1	
Use during the past 12 months: Yes	6	37.5	5	45.5	*X^2^* = 0.17; *df* = 1	
Age of onset	16.0 ± 2.0		16.5 ± 3.0		*t*(24) = 0.42	0.2
Current psychological treatment: Yes	5	23.8	3	14.3	*X^2^* = 0.61; *df* = 1	
Current psychiatric treatment: Yes	18	85.7	1	4.8	*X^2^* = 27.77^***^; *df* = 1	
Antidepressant medication	9	42.9	1	4.8	*X^2^* = 8.40^**^; *df* = 1	
Anxiolytic medication	5	23.8	0		*X^2^* = 5.67^*^; *df* = 1	
Anticonvulsant medication	5	23.8	0		*X^2^* = 5.67^*^; *df* = 1	
Antipsychotic medication	8	38.1	0		*X^2^* = 9.88^**^; *df* = 1	
Current main reason for consulting a mental health service:					*X^2^* = 5.60; *df*; 2	
Thought problems/suspiciousness or delusions	11	55.0	0			
Sadness/depression/anxiety	7	35.0	2	9.5		
Other reason/suicide attempt/fibromyalgia/grief	2	10.0	2	9.5		
Duration of untreated illness (DUI) (weeks)	118.7 ± 143.1		NA			
Psychological treatment in the past: Yes	15	71.4	17	81.0	*X^2^* = 0.52; *df* = 1	
Main reason for first psychological treatment:					*X^2^* = 3.22; *df* = 3	
Family/school problems	4	26.7	8	47.1		
Sadness/depression/anxiety	8	53.3	4	23.5		
Thought problems/suspiciousness or delusions	1	6.7	1	5.9		
Other/sexual abuse/PTSD	2	13.3	4	23.5		
Age at first psychological treatment (years)	12.8 ± 6.0		19.0 ± 7.5		*t*(30) = 2.52^**^	0.1
Psychiatric treatment in the past: Yes	6	28.6	3	14.3	*X^2^* = 2.50; *df* = 2	
Main reason for first psychiatric treatment:					*X^2^* = 2.25; *df* = 1	
Depression/anxiety	3	50.0	3	100		
Other/ADHD/suicide attempt	3	50.0	0			
Age at first psychiatric treatment	15.8 ± 8.9		18.6 ± 16.0		*t*(7) = 0.35	0.2
Previous psychiatric hospitalizations: Yes	3	14.3	0		*X^2^* = 3.23; *df* = 1	

ARMS, at-risk mental state; FHR, familial high risk; PTSD, post-traumatic stress disorder; ADHD, attention deficit hyperactivity disorder; and NA, not applicable. Mean ± *SD*.

*** *p* < 0.001;

** *p* < 0.01;

* *p* < 0.05.

Participants with an ARMS received the first psychological treatment at an earlier age than those with an FHR. In the ARMS group, the main reason for requesting this service was sadness, depression, or anxiety, whereas in the FHR group, it was family or school problems. No differences between groups were found in the age of first psychiatric treatment. In both groups, the main reason for requesting this service was depression and anxiety. In the ARMS group, 14.3% of participants had been hospitalized in a psychiatric institution for attempted suicide and/or anxiety (Table 2).

### Clinical and Functional Characteristics

As shown in Table 3, the ARMS group showed significantly higher scores with a large effect size than the FHR group on all clinical measures, which indicates greater severity of prodromal symptoms, schizotypal personality traits, and general psychopathology in the ARMS group as compared with the FHR group.

**Table 3:** Description and comparison of clinical measures between ARMS and FHR groups (*N* = 42).

	Possible range	ARMS (*n* = 21)	FHR (*n* = 21)	Mean comparison *t*-value (*df*)	Effect size Cohen’s *d*
		Mean ± *SD*	Mean ± *SD*		
Prodromal symptoms (CAARMS)^a^					
Positive symptoms					
Severity	0–24	13.0 ± 3.6	2.6 ± 3.0	−9.98^***^ (40)	−3.1
Frequency	0–24	12.0 ± 2.9	2.1 ± 2.5	−11.64^***^ (40)	−3.6
Cognitive change					
Severity	0–12	4.3 ± 1.8	1.9 ± 1.3	−4.88^***^ (40)	−1.5
Frequency	0–6	3.1 ± 1.4	1.9 ± 1.3	−2.60^*^ (40)	−0.9
Emotional disturbance					
Severity	0–18	6.1 ± 3.0	1.6 ± 2.5	−5.20^***^ (40)	−1.6
Frequency	0–18	6.9 ± 3.6	1.5 ± 2.3	−5.76^***^ (40)	−1.8
Negative symptoms					
Severity	0–18	7.5 ± 2.9	2.4 ± 2.3	−6.12^***^ (40)	−1.9
Frequency	0–18	8.6 ± 4.0	2.9 ± 3.2	−5.09^***^ (40)	−1.6
Behavioral change					
Severity	0–24	9.2 ± 3.5	2.2 ± 2.8	−6.98^***^ (40)	−2.2
Frequency	0–24	10.1 ± 3.9	6.5 ± 4.7	−1.58 (19)	−0.8
Motor/physical changes					
Severity	0–24	4.3 ± 2.6	1.1 ± 1.9	−4.42^***^ (40)	−1.4
Frequency	0–18	3.9 ± 2.6	0.9 ± 1.5	−4.49^***^ (40)	−1.4
General psychopathology					
Severity	0–48	14.0 ± 6.0	6.1 ± 4.1	−4.88^***^ (40)	−1.5
Frequency	0–48	15.1 ± 6.6	5.2 ± 4.1	−5.74^***^ (40)	−1.8
Schizotypal personality traits (SPQ)^a^	0–74	41.3 ± 16.3	16.3 ± 11.9	−5.33^***^ (35)	−1.7
Ideas of reference	0–9	4.4 ± 2.8	1.1 ± 1.7	−4.24^***^ (35)	−1.4
Magical thinking	0–7	2.2 ± 1.9	0.95 ± 1.5	−2.16^*^ (35)	−0.8
Unusual perceptual experiences	0–9	4.0 ± 3.1	1.4 ± 1.8	−2.99^**^ (35)	−1.0
Suspiciousness	0–8	4.2 ± 2.7	1.1 ± 1.4	−4.31^***^ (35)	−1.4
Excessive social anxiety	0–8	5.4 ± 2.4	3.2 ± 2.2	−2.90^**^ (35)	−0.1
No close friends	0–9	6.0 ± 2.4	2.8 ± 2.3	−4.07^***^ (35)	−1.4
Constricted affect	0–8	5.3 ± 2.0	1.6 ± 1.8	−5.75^***^ (35)	−1.9
Eccentric behavior	0–7	4.3 ± 1.7	1.1 ± 1.7	−5.73^***^ (35)	−1.9
Odd speech	0–9	4.4 ± 1.7	1.1 ± 1.7	−5.73^**^ (35)	−1.9
General psychopathology (SCL-90)^a^					
Somatization	0–4	1.5 ± 0.9	0.5 ± 0.6	−3.70^***^ (35)	−1.3
Obsession-Compulsion	0–4	2.3 ± 0.8	1.1 ± 0.7	−4.47^***^ (35)	−1.6
Interpersonal sensitivity	0–4	1.9 ± 0.9	0.8 ± 0.6	−4.42^***^ (35)	−1.4
Depression	0–4	0.1 ± 0.9	0.9 ± 0.6	−2.76^**^ (34)	−1.0
Anxiety	0–4	2.0 ± 1.1	0.7 ± 0.5	−4.17^***^ (35)	−1.5
Hostility	0–4	1.6 ± 1.1	0.4 ± 0.3	−4.39^***^ (35)	−1.0
Phobic anxiety	0–4	1.4 ± 1.0	0.3 ± 0.3	−4.01^***^ (34)	−1.5
Paranoid thoughts	0–4	1.8 ± 1.1	0.5 ± 0.6	−3.94^***^ (35)	−1.5
Psychotic	0–4	1.6 ± 1.0	0.4 ± 0.4	−4.70^***^ (35)	−1.6

ARMS, at-risk mental state; FHR, familial high risk; CAARMS, Comprehensive Assessment of At-Risk Mental States; SPQ, Schizotypal Personality Questionnaire; and SCL-90, Symptom Checklist-90.

a Higher score indicates greater severity of symptoms.

* *p* < 0.05;

** *p* < 0.01;

*** *p* < 0.001.

Descriptions and comparisons of premorbid adjustment and current functioning are shown in Table 4. According to the PAS score, the ARMS group reported more premorbid adjustment deficit than the FHR group in early adolescence (*p* = 0.028), late adolescence (*p* = 0.001), and adulthood (*p* = 0.002), with large effect sizes. As regards current overall social and role functioning, as expected, the ARMS group showed significantly lower scores on all measures of functioning than the FHR group, with large effect sizes.

**Table 4:** Description and comparison of premorbid and current functioning between ARMS and FHR groups.

	Possible range	ARMS (*n* = 21)	FHR (*n* = 21)	Mean comparison *t*-value (*df*)	Effect size Cohen’s *d*
		Mean ± *SD*	Mean ± *SD*		
Premorbid adjustment (PAS)^a^					
Childhood	0–1	0.3 ± 0.2	0.2 ± 0.1	−1.67 (40)	−1.0
Early adolescence	0–1	0.4 ± 0.2	0.2 ± 0.1	−2.27^*^ (40)	−1.3
		*n* = 17	*n* = 21		
Late adolescence^b^	0–1	0.5 ± 0.2	0.2 ± 0.1	−4.57^***^ (37)	−1.9
		*n* = 14	*n* = 20		
Adulthood^c^		0.5 ± 0.2	0.2 ± 0.1	−3.13^**^ (31)	−1.9
Current social functioning					
Social and occupational functioning (SOFAS)^d^	0–100	58.1 ± 7.9	84.1 ± 7.8	10.67^***^ (40)	3.3
GF-Social^d^	0–10	5.7 ± 1.1	8.4 ± 0.8	9.13^***^ (40)	2.8
GF-Role^d^	0–10	6.4 ± 1.2	8.4 ± 0.8	6.44^***^ (40)	1.9

ARMS, at-risk mental state; FHR, familial high risk; PAS, Premorbid Adjustment Scale; and SOFAS, Social and Occupational Functioning Assessment Schedule: GF-Social, GF-Role.

a Lower score indicates “healthier” level of functioning.

b Late adolescence subscale of PAS was not applicable for patients under 15.

c Adult subscale of the PAS was not applicable for patients under 18.

d Higher scores indicate greater levels of functioning.

* *p* < 0.05;

** *p* < 0.01;

*** *p* < 0.001.

## Discussion

To the best of our knowledge, this is the first study in Mexico to describe and compare the sociodemographic, clinical, and functional characteristics of ARMS youths and first- or second-degree relatives of patients diagnosed with a psychotic spectrum disorder. Overall, the ARMS group was significantly younger, had fewer years of schooling, and were more likely to be male than those in the FHR group. The ARMS group showed greater severity of prodromal symptoms, schizotypal personality traits, general psychopathology, and lower levels of premorbid and current functioning than the FHR group.

Consistent with Fusar-Poli et al. (2020), the ARMS group showed similar demographic characteristics, such as being young, predominantly male, and single. Moreover, most of the ARMS participants had completed middle school and were still at school when they took part in the study. Similar data were found by Kotlicka-Antczak et al. (2018) in Polish ARMS individuals, although unlike ours, their sample had a slight predominance of female participants.

In contrast to He et al. (2021), participants in our study with an FHR did not differ in marital status from those with an ARMS. According to Terzian et al. (2007), offspring of people with schizophrenia have social adjustment problems in their adult lives that are reflected in their marital status and employment and should therefore be identified as a vulnerable group.

The average age at onset of cannabis use (18 years) was slightly higher than that found in a recent meta-analysis (<18 years of age; Farris et al., 2020). However, our findings are consistent with estimates of the percentage of ARMS individuals who use or have used cannabis at least once in their lifetime (Farris et al., 2020).

It is striking that the ARMS individuals received their first psychological treatment in early adolescence for depressive or anxiety symptoms, which is consistent with the fact that most mental disorders emerge before the age of 25 with 50% of patients already being symptomatic by the age of 14 (Kessler et al., 2005). In addition, quantitative and qualitative research has suggested that anxiety and depressive symptoms frequently mark the onset of the initial prodrome of psychosis (Hafner et al., 1999; Corcoran et al., 2007). These symptoms were one of the main reasons why those in the ARMS group have continued to seek help in their current psychological or psychiatric treatment.

Those in the FHR group received their first psychological treatment in adulthood for family problems. During this period in their lives, they probably needed professional support because they began to be more aware of the mental illness of their parents or siblings, its implications for the family environment, and their role as potential caregivers. Boström and Strand (2021), in a qualitative study, have suggested that the children of people with psychosis may have an unclear picture of their parents’ illness during childhood and early adolescence (8- to 15-year-old), even if they recall having been informed of the illness by their parents or by mental health services. It seems that parents often avoid discussing details of their mental illness with their children to protect them from the associated stigma. In addition, studies report that siblings of people with psychosis may develop survivor guilt and experience long-standing grief at the loss of the personality of their sick sibling and a lack of understanding of their illness (Bowman et al., 2014).

As expected, our findings indicate major clinical differences between the groups. ARMS participants showed greater severity not only of psychotic symptoms and schizotypal personality but also of general psychopathology than the FHR group. The mean CAARMS scores from other studies (Domínguez-Martínez et al., 2017; Pelizza et al., 2019) are like those of the ARMS group in the present study. In addition, studies have noted the high psychopathological heterogeneity in ARMS individuals (Addington et al., 2020), particularly in depressive and anxiety symptoms (Fusar-Poli et al., 2014). Moreover, several researchers have highlighted the presence of a significant proportion of non-psychotic psychiatric comorbidity among ARMS people fulfilling the criteria for both ARMS and at least one non-psychotic illness (Salokangas et al., 2012; Hui et al., 2013; Fusar-Poli et al., 2014). Although the FHR group obtained significantly lower general psychopathology scores than the ARMS group, their symptom severity levels are higher than those of subjects in studies of healthy Mexican populations (Ramírez and Martínez, 2016; Martínez et al., 2020), particularly in the dimensions of obsession-compulsion, hostility, depression, and anxiety. Previous research has underlined the high prevalence of depression and anxiety among unaffected first- and second-degree relatives of people with psychosis, and it has even been suggested that having this kinship with a person with psychosis could be a risk factor for the development of any mental disorder, not just for psychosis (Shah et al., 2019). Although clinical high-risk symptoms were absent in the FHR group, it is important to consider that they already showed various types of psychopathology that require attention, especially since they are a population at risk of developing psychosis due to their genetic vulnerability. It is important for future research to prospectively explore larger samples of at FHR young people with and without subthreshold psychotic symptoms to better understand why some at FHR individuals develop subthreshold symptoms and others do not (Stowkowy and Addington, 2013).

We also found that participants with FHR showed stable premorbid functioning across the stages of development, whereas ARMS participants showed significantly lower levels of premorbid functioning from early adolescence to adulthood. This is consistent with previous studies that found lower levels of premorbid functioning in ARMS individuals as compared to those of FHR or healthy controls (Stowkowy and Addington, 2013; Dannevang et al., 2018). These early functional difficulties should be the target of timely preventive strategies since they constitute the first signs of psychosis risk before ARMS symptoms emerge that could be susceptible to change if effective interventions are provided (Fiorillo, 2019). Finally, in line with previous research (Fusar-Poli et al., 2014), the ARMS group in the current study showed greater impairment in social and occupational functioning than the FHR group. This finding is important because there is evidence that a significant percentage of ARMS individuals continue to function poorly in the long term, regardless of symptomatic remission (Addington et al., 2011). In a recent qualitative study, Cotter et al. (2019) found that ARMS individuals attributed their impairment in social and occupational functioning to a combination of clinical, cognitive, and psychological factors, such as self-stigmatizing attitudes and dysfunctional metacognitive beliefs. It is important to note that the FHR group showed a good level of functioning, which confirms that a family history of psychosis is a significant risk factor but not an impediment for certain people to be able to perform adequately.

This study has certain limitations that should be borne in mind. First, sample size is small and, therefore, findings should be interpreted with caution. Second, participants were recruited from a specialized tertiary care psychiatric hospital that usually attends more severe patients than primary care services. Nevertheless, overall findings provide richly detailed information on the clinical and functional characteristics of Mexican ARMS individuals, and differences from FHR, which can help visibilize a population that has received scant attention in mental health services in developing countries.

Given the complex etiology and heterogeneous clinical manifestation of psychosis, it is important for research to better characterize the UHR stage to improve early detection and arrive at a valid cross-cultural definition of the at-risk mental state in different populations (Fridgen et al., 2013). Most early psychosis studies have been conducted in high-income countries or low- and middle-income countries in Asia and Africa (Cohen et al., 2008). However, this line of research is extremely limited in Latin America, where more regionally adapted knowledge is needed to improve the planning of specialized early psychosis services that are almost nonexistent (Nicolini, 2009; Brietzke et al., 2011). Given the lack of studies focusing on the pre-psychotic stage in Mexico and Latin America, the improvement of early detection strategies and preventive interventions at the early stages of psychosis should be a priority to reduce the duration of untreated psychosis, close the healthcare gap, and lower long-term mental health costs. In addition, future research should focus on assessing transition rates and, importantly, risk factors associated with the transition to psychosis.

## Data Availability Statement

The raw data supporting the conclusions of this article will be made available by the authors, without undue reservation.

## Ethics Statement

The studies involving human participants were reviewed and approved by the Comité de Etica en Investigación. National Institute of Psychiatry Ramón de la Fuente Muñiz. Written informed consent to participate in this study was provided by the participants’ legal guardian/next of kin.

## Author Contributions

LN and TD-M: conceptualization, formal analysis, methodology, data collection, writing—original draft, and writing—review and editing. MR-V, RS-A, CC-B, and MR-G reviewed and approved the final version of the manuscript. All authors contributed to the article and approved the submitted version.

## Funding

This study was supported by the Mexican National Council for Science and Technology (Consejo Nacional de Ciencia y Tecnología, CONACyT), grant no. A1-S-21384.

## Conflict of Interest

The authors declare that the research was conducted in the absence of any commercial or financial relationships that could be construed as a potential conflict of interest.

## Publisher’s Note

All claims expressed in this article are solely those of the authors and do not necessarily represent those of their affiliated organizations, or those of the publisher, the editors and the reviewers. Any product that may be evaluated in this article, or claim that may be made by its manufacturer, is not guaranteed or endorsed by the publisher.
